# Characteristics of a trauma population in an ambulance organisation in Sweden: results from an observational study

**DOI:** 10.1186/s13049-023-01090-0

**Published:** 2023-06-26

**Authors:** Glenn Larsson, Christer Axelsson, Magnus Andersson Hagiwara, Johan Herlitz, Carl Magnusson

**Affiliations:** 1 PreHospen-Centre for Prehospital Research, Faculty of Caring Science, Work Life and Social Welfare, University of Borås, Allegatan 1, 501 90 Borås, Sweden; 2 Department of Prehospital Emergency Care, Sahlgrenska University Hospital, Gothenburg, Sweden; 3 Department of Molecular and Clinical Medicine, Institute of Medicine, Sahlgrenska Academy, University of Gothenburg, Gothenburg, Sweden

**Keywords:** Trauma, Injury, Emergency medical service, Ambulance services, Patient triage, Mortality

## Abstract

**Background:**

Globally, injuries are a major health problem, and in Sweden, injuries are the second most common reason for ambulance dispatch. However, there is a knowledge gap regarding the epidemiology of injuries requiring assessment by emergency medical services (EMS) in Sweden. The aim of the present study was to describe the prehospital population with injuries that have been assessed and treated by EMS.

**Methods:**

A randomly selected retrospective sample was collected from 1 January through 31 December 2019 in a region in southwestern Sweden. Data were collected from ambulance and hospital medical records.

**Results:**

Among 153,724 primary assignments, 26,697 (17.4%) were caused by injuries. The study cohort consisted of 5,235 patients, of whom 50.5% were men, and the median age was 63 years. The most common cause of injury was low-energy fall (51.4%), and this was the cause in 77.8% of those aged > 63 years and in 26.7% of those aged ≤ 63 years. The injury mechanism was a motor vehicle in 8.0%, a motorcycle in 2.1% and a bicycle in 4.0%. The most common trauma location was the residential area (55.5% overall; 77.9% in the elderly and 34.0% in the younger group). In the prehospital setting, the most frequent clinical sign was a wound (33.2%), a closed fracture were seen in 18.9% and an open fracture in 1.0%. Pain was reported in 74.9% and 42.9% reported severe pain. Medication was given to 42.4% of patients before arrival in the hospital. The most frequent triage colour according to the RETTS was orange (46.7%), whereas only 4.4% were triaged red. Among all patients, 83.6% were transported to the hospital, and 27.8% received fracture treatment after hospital admission. The overall 30-day mortality rate was 3.4%.

**Conclusion:**

Among EMS assignments in southwestern Sweden, 17% were caused by injury equally distributed between women and men. More than half of these cases were caused by low-energy falls, and the most common trauma location was a residential area. The majority of the victims had pain upon arrival of the EMS, and a large proportion appeared to have severe pain.

## Introduction

Globally, injuries are a major health problem. According to the World Health Organization [1], injuries accounted for 9% of all deaths in the world and 12% of all health problems in 2000. Most injury-related deaths occur in low- and middle-income countries, and men account for the majority of deaths. Injuries caused by traffic accidents are the major cause. People who die from injuries are relatively young; almost half are between 15 and 44 years old [1]. In 2013, 916 million people were injured, and 56 million needed hospital care. Furthermore, more than 66 million people die from their injuries [2]. Various types of transport accidents cause almost 10% of all injuries. Other major causes of injuries were the impact of mechanical forces and fall accidents [2]. In that study a majority died prehospitally.

The epidemiology of injury differs globally from that of Nordic countries. A Norwegian study [3] showed that among people of working age, transport accidents accounted for 30% of all injury-related deaths, and fall accidents accounted for 8.1%. Self-inflicted injury was the most common cause of injury-related death. This differs markedly from the global figure, where self-inflicted injuries accounts for less than half as much of injury-related deaths [2]. Another Norwegian study [4] reported that 30-day mortality was significantly higher among patients with injuries over 64 years of age. In addition, the type of injury differed by age, and the proportion of head injuries and injuries in the pelvis and lower extremities increased with age. The mechanism of injury also differed between the age groups. Low-energy falls account for the most common mechanism among patients over 64 years [4].

In 2020, 141,000 people were admitted to Swedish hospitals due to various types of injuries. The most common type of injury was fall-injuries, which accounted for 70% of all hospitalised patients. Among them, the majority were over 65 years. In 6% of cases, the cause of hospital care was road-traffic injury, twice as many men as women [5].

Injury is the second most common reason for ambulance dispatch in Sweden [6]. Minor injuries are probably the most common, since 15% of all these cases are not transported to the hospital, but today, there is a knowledge gap regarding the prehospital population assessed and treated by the EMS due to injuries. Most likely, prehospital assessment and treatment are important in the care process for injured patients. Both prehospital time and quality of care are important variables for patients with injuries. Prehospital mortality following injury is higher in rural areas than in urban areas, but hospital mortality does not differ between these two groups [7]. This suggests that time to prehospital care and time to hospital after the accident may be important factors in the outcome. Maybe can physician-staffed prehospital intensive care teams increase survival rates for patients with serious injuries [8]. In order to be able to prepare the prehospital organisation with resources, training and decision-making, it is important to map the prehospital population that calls for EMS due to injuries. Against this background, the present study aims to describe the prehospital population with injuries that have been assessed and treated by EMS.

## Methods

### Study design

In this retrospective observational study, the epidemiology of trauma patients was investigated by reviewing EMS and hospital medical records. The model for methods in chart review studies suggested by Kaji et al. (2014) was used as a guide for the study design and methodology [9].

### Settings and population

The study was conducted in a region in southwestern Sweden that has approximately 1.7 million inhabitants with the following age distribution: 56% is under 45 years of age, 24% are between 45 and 64 years old and 19% are over 65 years old. The average life expectancy for women is 84.0 years and for men 80.3 years. The region has an area of 23,942 km² and a population density of 73/km². The majority of the region’s inhabitants live in cities, but the region also has some rural areas that are relatively far from the nearest hospital. The region has five hospital administrations and 10 hospitals with emergency departments (EDs) [10]. The EMS organisation in the region is divided into five hospital administrations and has 46 ambulance stations spread across the region, with approximately 110 ambulance units. In 2019, 173,536 ambulance assignments were carried out in the region [11].

The ambulances in the examined region are staffed with one or two registered nurses (RNs). Most have a one-year postgraduate education in prehospital emergency care at the master’s level. In addition to the RN, the other crew member is an emergency medical technician (EMT) with assistant nurse training, as well as 40-week supplementary training in prehospital emergency care [12]. The emergency medical dispatch centre (EMDC) in the region assesses the urgency of ambulance assignments, assigns the appropriate priority level and dispatches the ambulances from three levels: Priority 1 (life-threatening, lights and sirens), Priority 2 (urgent, but not life threatening) and Priority 3 (can wait but in need of ambulance transport).

RNs in the region’s ambulances can independently administer about 30 different medicines according to regional guidelines. These guidelines describe processes for patient assessment and treatment of various symptoms and conditions. In addition to the general guidelines, there are protocols that describe specific care processes that may differ across the region [13]. All EMS organisations in the region at the time of the study used the Rapid Emergency Triage and Treatment System (RETTS) [14] licenced and maintained by Predicare AB. The triage system is a five-level system, and the patient in the prehospital setting at the scene is assessed with a colour that determines how acute their condition is. The definitions of the colours are as follows: red, ‘life-threatening’; orange, ‘potentially life-threatening’; yellow, ‘non-life-threatening’; green, ‘non-life-threatening and not in need of immediate care’; and blue, ‘no need for triage’. The triage system is based on two variables, vital signs (VS) and emergency signs and symptoms (ESS), both of which yield a colour and thus represent a level of severity. The highest colour of either VS or ESS becomes the final triage level. Red and orange levels are considered direct acute care processes, whereas yellow and green can wait in the emergency department (ED) without jeopardising the individual medical risk.

### Data sampling

**Fig. 1:**
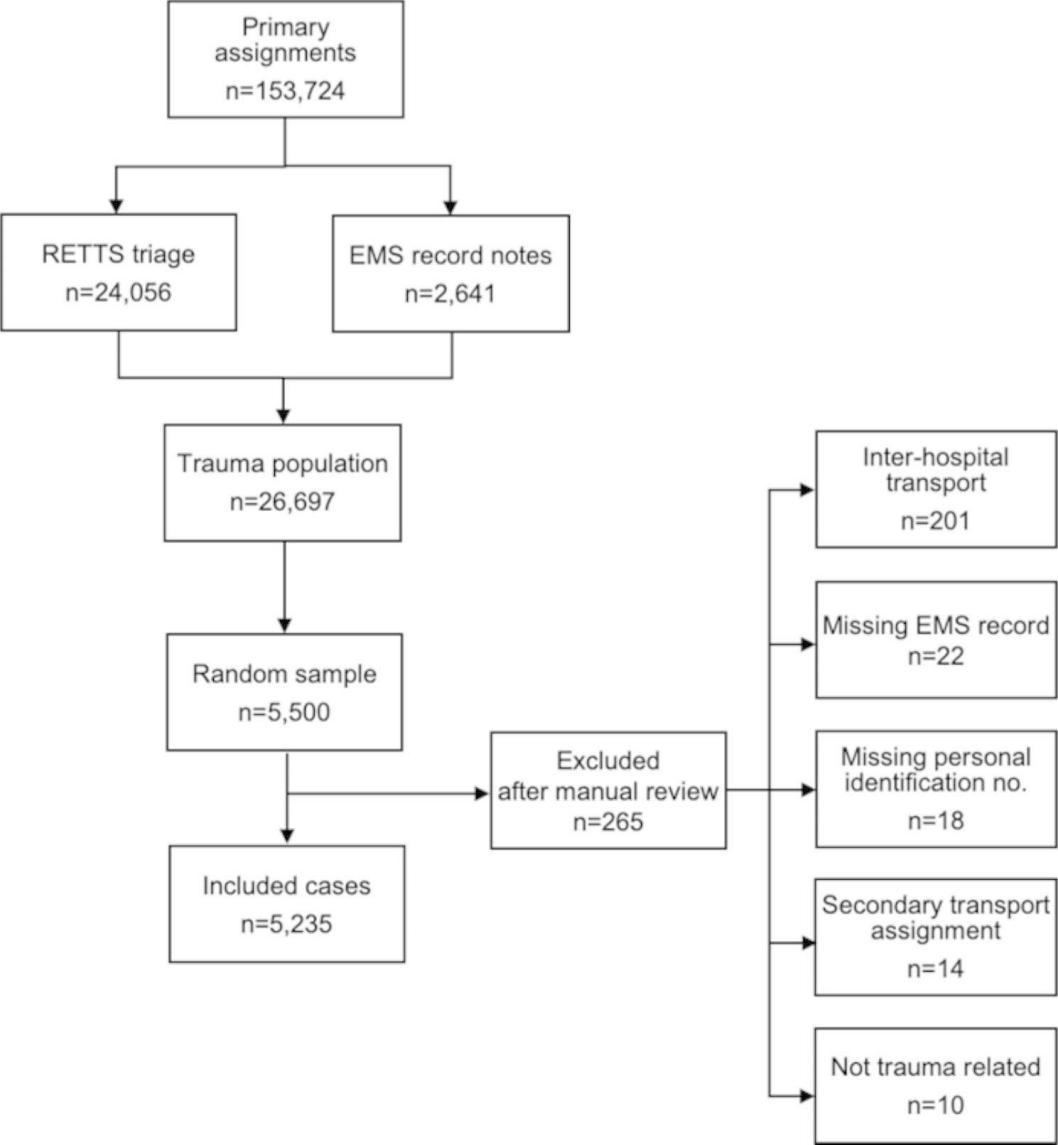
Flowchart of the inclusion

A retrospective sample was collected from 1 January to 31 December 2019. The inclusion criteria were primary assignments registered with a contact reason that indicated any type of injury or physical trauma. A total of 153,724 primary assignments were conducted in 2019. Out of the total number of primary assignments, cases related to trauma were selected, which comprised both RETTS and ESS codes indicating a trauma (n = 24,056) and assignments where no RETTS had been registered (n = 2,641). The latter was included in order not to overlook any potential time-critical patients, where there may be no time to use the triage system in the prehospital setting. A total of 26,697 records were found to be related to trauma and comprised 17.4% of the total annual primary assignments. In the second phase, a random sample was drawn from the total number of identified trauma assignments. A sample of 5,500 EMS records (20.6%) was drawn based on assignments and proportional distribution from each of the five EMS organisations to receive a representative sample of the total trauma population. After manual review by one designated ambulance RN in each of the five EMS organisations in the region, 265 assignments were excluded, and a total of 5,235 patients were included (Fig. 1).

Data for the included assignments were collected from the EMS electronic patient journal system and electronic hospital records. From prehospital records, the following data were retrieved: ambulance assignment number, patient personal identification number, time and date, sex, age, priority from dispatch centre, site of injury, vital parameters, RETTS and ESS codes, triage colour, type of injury, mechanism of injury, blunt or penetrating trauma, treatment, assessment of pain intensity according to numeric rating scale (NRS), transport mode as ground ambulance, patient transport, helicopter emergency medical services (HEMS), police vehicle, decision on conveyance to hospital, referral to self-care or primary care, renewed ambulance assignment within 72 h and transport destination. The hospital’s electronic record system provided data on past medical history, admission to hospital, treatment at hospital, length of hospital stays and discharge destination from hospital. Information on mortality after 2, 7 and 30 days was retrieved from the Swedish population registry.

### Analysis

The descriptive statistics were calculated of the random sample (5500 cases). In the tables, the data are presented as numbers and percentages, with central tendencies of dispersion when applicable. The available data were used in the calculations. Patients were grouped into low or high acuity groups and by median age in the cohort (≤ 63 years of age and over 63 years of age. Patients assessed at the scene by the EMS RN with RETTS triage levels of blue, green and yellow were defined as low acuity, whereas triage levels orange and red were defined as high acuity. Two-group comparisons were performed with Pearson’s chi-squared, Fisher’s exact test or the Wilcoxon rank-sum test where applicable. All tests were two-sided, and p-values below 0.01 were considered significant. Data processing and statistical analysis were performed with RStudio version 2022.7.2.576 (RStudio: Integrated Development Environment for R. RStudio, PBC, Boston, MA URL http://www.rstudio.com/) and with the package tbl_summary (Daniel D.Sjöberg et al.).

### Ethics

This study was approved by the Swedish Ethical Review Authority in Stockholm, Sweden (Dnr 2020 − 00490). Prior to the journal review, approval was obtained from the heads of operations of the participating organisations. The project received an approved ethics review without informed consent from patients whose records had been reviewed. The motivation is that a record review is a common method for healthcare organisations to measure patient safety and quality of care. The approach in this research project does not differ from the journal review that normally takes place within healthcare organisations. People employed by the included organisations executed the journal reviews. The study ensured strict compliance with Swedish research ethics guidelines [15].

## Results

### Patients with injury stratified by sex, age and health history

Among the patients with injuries assessed and treated by the EMS in 2019, there were slightly more men than women. The median age of the patients was 63 years, and the largest age group was between 18 and 64 years (41.3%). There were more women in the two oldest age groups of 65–79 and over 80 years, 20.3% vs. 19.0% and 37.6% vs. 20.7% respectively, whereas men were more common in the age groups 0–17 and 18–64 years of age (12.1% vs. 7.9% and 48.2% vs. 34.2%), p < 0.001. The most common previous diseases were various types of cardiovascular disease, where hypertension was predominant (38.8%). Women were found to have more circulatory diagnoses (43.2% vs. 34.7%, p < 0.001), and a majority (69%) of all patients over 63 years of age were linked to previous diseases of circulatory origin. Past medical history of a psychiatric diagnosis was the second most common previous history group and was more common among women and patients older than 63 years of age, 23.1% vs. 20% (p = 0.008) and 23.2% vs. 19.8% (p = 0.004) respectively (Table 1).

**Table 1:** Patient age, sex and past medical history stratified on sex and median age

	N = 5,235^*1*^	Male, N = 2,610^*1*^	Female, N = 2,561^*1*^	p-value^*2*^	≤ 63 yrs, N = 2,613^*1*^	> 63 yrs, N = 2,567^*1*^	p-value^*2*^
Age in years (55)^3^	63 (33, 82)	54 (28, 77)	72 (42, 86)	< 0.001	33 (20, 51)	82 (74, 88)	< 0.001
Age Groups (55)				< 0.001			< 0.001
0–17	522 (10.1)	314 (12.1)	202 (7.9)		522 (20.0)	0 (0.0)	
18–64	2,140 (41.3)	1,256 (48.2)	875 (34.2)		2,091 (80.0)	49 (1.9)	
65–79	1,016 (19.6)	495 (19.0)	520 (20.3)		0 (0.0)	1,016 (39.6)	
≥ 80	1,502 (29.0)	539 (20.7)	962 (37.6)		0 (0.0)	1,502 (58.5)	
Male sex (64)	2,610 (50.5)				1,539 (59.2)	1,065 (41.5)	< 0.001
Diseases of the circulatory system (269)	1,928 (38.8)	855 (34.7)	1,066 (43.2)	< 0.001	223 (9.0)	1,700 (69.0)	< 0.001
Mental, Behavioral and Neurodevelopmental disorders	1,066 (21.5)	492 (20.0)	570 (23.1)	0.008	491 (19.8)	572 (23.2)	0.004
Endocrine, nutritional and metabolic diseases	473 (9.5)	261 (10.6)	210 (8.5)	0.013	90 (3.6)	381 (15.5)	< 0.001
Diseases of the respiratory system	388 (7.8)	158 (6.4)	230 (9.3)	< 0.001	103 (4.2)	285 (11.6)	< 0.001
Neoplasms	314 (6.3)	163 (6.6)	149 (6.0)	0.4	32 (1.3)	280 (11.4)	< 0.001
Diseases of the musculoskeletal system and connective tissue	155 (3.1)	30 (1.2)	125 (5.1)	< 0.001	43 (1.7)	112 (4.5)	< 0.001
Diseases of the genitourinary system	129 (2.6)	82 (3.3)	46 (1.9)	0.001	13 (0.5)	115 (4.7)	< 0.001
Other condition	2,217 (44.6)	975 (39.6)	1,233 (50.0)	< 0.001	703 (28.4)	1,506 (61.1)	< 0.001

^*1*^Median (IQR); n (%) ^*2*^Wilcoxon rank sum test; Pearson’s Chi-squared test  ^3^Missing data

### Cause and place of injury

The most common cause of injury was low-energy falls (51.4%), and the majority of these patients were over 63 years old (77.8% vs. 26.75, p < 0.001). High-energy falls were also a relatively common reason for injury (9.5%), but in those cases, the majority of victims were under 63 years (12.5% vs. 6.4%, p < 0.001).The most common place where these injuries occurred was in the patients’ homes (55%). Patients over 63 years of age were overrepresented when the trauma took place in the patient’s home (77.9% vs. 34.0%, p < 0.001), while injuries occurring in public places were more common in patients under 63 years of age (44.9% vs. 18.5%, p < 0.001). Patients with low-energy falls were the largest proportion assessed as life-threatening or potentially life-threatening (i.e., high acuity) (58.5% vs. 48.6%, p < 0.001). In total, traffic accidents were few, and the percentage of high acuity in this group was relatively low compared with injuries not related to traffic in total (13.1% vs. 86.7%). Table 2).

**Table 2:** Injury mechanism and trauma location in relation to prehospital assessed severity and median age

	N = 5,235^*1*^	Low acuity, N = 2,315^*1*^	High acuity, N = 2,428^*1*^	p-value^*2*^	≤ 63 yrs, N = 2,613^*1*^	> 63 yrs, N = 2,567^*1*^	p-value^*2*^
Injury mechanism (184)^3^							
Motor vehicle	407 (8.1)	195 (8.7)	129 (5.5)	< 0.001	347 (13.6)	51 (2.1)	< 0.001
Motorcycle	106 (2.1)	22 (1.0)	63 (2.7)	< 0.001	100 (3.9)	5 (0.2)	< 0.001
Bicycle	200 (4.0)	89 (4.0)	92 (3.9)	> 0.9	155 (6.1)	41 (1.7)	< 0.001
Pedestrian	20 (0.4)	12 (0.5)	5 (0.2)	0.072	16 (0.6)	3 (0.1)	0.004
Other traffic accident	40 (0.8)	18 (0.8)	18 (0.8)	0.9	27 (1.1)	12 (0.5)	0.022
Shot by firearm	10 (0.2)	1 (0.0)	6 (0.3)	0.13	8 (0.3)	1 (0.0)	0.039
Stabbed by knife or other sharp object	201 (4.0)	99 (4.4)	67 (2.9)	0.005	178 (7.0)	17 (0.7)	< 0.001
Struck or hit by blunt object	389 (7.7)	222 (9.9)	126 (5.4)	< 0.001	348 (13.7)	31 (1.3)	< 0.001
Low energy fall	2,598 (51.4)	1,089 (48.6)	1,374 (58.5)	< 0.001	680 (26.7)	1,909 (77.8)	< 0.001
High energy fall	478 (9.5)	200 (8.9)	234 (10.0)	0.2	318 (12.5)	158 (6.4)	< 0.001
Blast injury	3 (0.1)	1 (0.0)	1 (0.0)	> 0.9	3 (0.1)	0 (0.0)	0.3
Other injury	599 (11.9)	294 (13.1)	233 (9.9)	< 0.001	368 (14.4)	227 (9.2)	< 0.001
Trauma location (263)							
Residential area	2,758 (55.5)	1,150 (52.6)	1,394 (59.8)	< 0.001	845 (34.0)	1,903 (77.9)	< 0.001
Public area	1,597 (32.1)	713 (32.6)	692 (29.7)	0.034	1,116 (44.9)	453 (18.5)	< 0.001
Stable	57 (1.1)	22 (1.0)	31 (1.3)	0.3	53 (2.1)	4 (0.2)	< 0.001
Workplace	138 (2.8)	64 (2.9)	66 (2.8)	0.8	130 (5.2)	7 (0.3)	< 0.001
Leisure activity	273 (5.5)	161 (7.4)	92 (3.9)	< 0.001	255 (10.3)	13 (0.5)	< 0.001
School	31 (0.6)	22 (1.0)	4 (0.2)	< 0.001	31 (1.2)	0 (0.0)	< 0.001
Nursing home	35 (0.7)	8 (0.4)	24 (1.0)	0.008	4 (0.2)	31 (1.3)	< 0.001
Prison/detention centre	5 (0.1)	4 (0.2)	0 (0.0)	0.055	4 (0.2)	1 (0.0)	0.4
Nature/forrest	23 (0.5)	8 (0.4)	14 (0.6)	0.3	9 (0.4)	14 (0.6)	0.3
Water activity	14 (0.3)	5 (0.2)	6 (0.3)	0.8	11 (0.4)	3 (0.1)	0.035
Other location	56 (1.1)	37 (1.7)	17 (0.7)	0.003	37 (1.5)	18 (0.7)	0.012

^*1*^n (%) ^*2*^Pearson’s Chi-squared test; Fisher’s exact test ^*3*^Missing data

### Type of injury, symptoms and vital parameters during prehospital care

The three most common types of injury were wounds (33.2%), followed by haematoma, swelling, bruising or abrasion (21.4%) and fractures (18.9%). The most common signs and symptoms noted by EMS clinicians on site were paleness (5.9%), nausea (5.3%) and dizziness (3.3%). Cardiac arrest at the accident site was noted in 0.6% of the cases. The median values of the vital parameters were normal in the population. Patients over 63 years of age had lower oxygen saturation and higher blood pressure and serum glucose levels in comparison with patients under 63 years of age. Among the patients in whom the severity of pain was assessed with the NRS, 42.8% had a very high intensity Please, remove following ref from this resulttext [7–10]. However, only in a minority of patients (17.5%) was the intensity of pain assessed with the NRS. Patients under 63 years of age reported more severe pain than the elderly (50.9% vs. 37.1%) (Table 3).

**Table 3:** Prehospital clinical observations regarding type of injury, vital signs and pain intensity in relation to assessed severity and median age

	N = 5,235^*1*^	Low acuity, N = 2,315^*1*^	High acuity, N = 2,428^*1*^	p-value^*2*^	≤ 63 yrs, N = 2,613^*1*^	> 63 yrs, N = 2,567^*1*^	p-value^*2*^
Type of injury (132)^3^							
Wound	1,695 (33.2)	742 (32.8)	793 (33.1)	0.8	829 (32.5)	851 (33.9)	0.3
Pressure ulcer	22 (0.4)	9 (0.4)	13 (0.5)	0.5	4 (0.2)	18 (0.7)	0.002
Closed fracture	963 (18.9)	250 (11.0)	685 (28.6)	< 0.001	307 (12.0)	655 (26.1)	< 0.001
Open fracture	52 (1.0)	8 (0.4)	38 (1.6)	< 0.001	37 (1.5)	15 (0.6)	0.003
Dislocation	237 (4.6)	48 (2.1)	181 (7.5)	< 0.001	125 (4.9)	111 (4.4)	0.4
Observered burn	38 (0.7)	14 (0.6)	19 (0.8)	0.5	31 (1.2)	7 (0.3)	< 0.001
Hematoma/Swelling/ Bruising/Abrasion	1,093 (21.4)	543 (24.0)	483 (20.1)	0.002	510 (20.0)	571 (22.7)	0.018
Teeth injury	35 (0.7)	19 (0.8)	13 (0.5)	0.2	26 (1.0)	9 (0.4)	0.005
Oto/Rhino/Laryngeal/Orbital bleeding	36 (0.7)	16 (0.7)	16 (0.7)	0.9	24 (0.9)	12 (0.5)	0.050
Head bleeding	7 (0.1)	0 (0.0)	6 (0.3)	0.032	2 (0.1)	5 (0.2)	0.3
Suspicion of internal bleeding	5 (0.1)	1 (0.0)	4 (0.2)	0.4	0 (0.0)	5 (0.2)	0.030
Other injury/unharmed	1,719 (33.7)	908 (40.1)	604 (25.2)	< 0.001	996 (39.1)	704 (28.0)	< 0.001
Vital signs							
Respiratory rate/min (803)	18 (15,22)	18 (15,20)	18 (15,24)	< 0.001	18 (15,22)	18 (15,22)	0.5
Pulse rate/min (628)	85 (66,110)	85 (67,106)	84 (65,110)	0.4	89 (70,112)	80 (64,104)	< 0.001
Oxygen saturation % (651)	97 (93,100)	98 (95,100)	97 (92,99)	< 0.001	98 (95,100)	96 (92,99)	< 0.001
Systolic blood pressure mm/hg (937)	140 (110,170)	140 (112,170)	140 (110,171)	0.5	130 (110,160)	142 (115,180)	< 0.001
Body temperature ℃ (1615)	36.8 (36.0,37.5)	36.9 (36.0,37.5)	36.8 (36.0,37.5)	< 0.001	36.8 (36.0,37.5)	36.8 (36.0,37.5)	0.3
Glasgow coma scale (1209)	15 (15,15)	15 (15,15)	15 (15,15)	< 0.001	15 (15,15)	15 (15,15)	0.7
Blood glucose mmol/L (3778)	7.0 (5.1,11.4)	6.6 (4.9,10.8)	7.1 (5.2,11.5)	< 0.001	6.3 (4.8,9.6)	7.4 (5.4,11.8)	< 0.001
Pain yes (381)	3,636 (74.9)	1,568 (71.6)	1,893 (82.4)	< 0.001	1,754 (73.1)	1,864 (77.1)	0.001
Pain level NRS (4310)				0.002			< 0.001
0–3	282 (30.5)	110 (34.9)	165 (27.7)		90 (23.3)	192 (35.8)	
4–6	247 (26.7)	95 (30.2)	149 (25.0)		100 (25.8)	146 (27.2)	
7–10	396 (42.8)	110 (34.9)	282 (47.3)		197 (50.9)	199 (37.1)	

^*1*^n (%); Median (10%,90%) ^*2*^Pearson’s Chi-squared test; Fisher’s exact test; Wilcoxon rank sum test ^3^Missing data

### Prehospital assessments and treatments

The most common dispatch priority was Priority 2 (urgent but no lights or sirens) (63.1%). Patients under 63 years of age were more often given Priority 1 (lights and sirens) than the elderly (48.4% vs. 18.4%). Also in EMS priority, Priority 2 was the most common (68.0%), and the most common RETTS triage colour was orange (46.7%). In patients over the age of 63 it was more common with orange RETTS triage colour (55.5% vs. 37.7%), whereas in the RED triage group patients 63 years of age and below were more frequently triaged to RED compared to the older patients (6.3% vs. 2.7%). In all, 13.8% of the patients remained at the scene, and 74.8% were transported to the ED. Among all patients, 83.6% were transported to the hospital (Table 4).

**Table 4:** Emergency medical dispatch center (EMDC) priority, emergency medical services (EMS) triage, mode of transport and decision on destination in relation to assessed patient severity and age

	N = 5,235^*1*^	Low acuity, N = 2,315^*1*^	High acuity, N = 2,428^*1*^	p-value^*2*^	≤ 63 yrs, N = 2,613^*1*^	> 63 yrs, N = 2,567^*1*^	p-value^*3*^
EMDC Priority (3)^4^				< 0.001			< 0.001
1	1,771 (33.8)	658 (28.4)	853 (35.1)		1,262 (48.4)	473 (18.4)	
2	3,301 (63.1)	1,548 (66.9)	1,539 (63.4)		1,304 (50.0)	1,979 (77.1)	
3	160 (3.1)	109 (4.7)	36 (1.5)		44 (1.7)	115 (4.5)	
Triage level (492)				< 0.001			< 0.001
Red	211 (4.4)	0 (0.0)	211 (8.7)		143 (6.3)	66 (2.7)	
Orange	2,217 (46.7)	0 (0.0)	2,217 (91.3)		855 (37.7)	1,355 (55.5)	
Yellow	1,286 (27.1)	1,286 (55.6)	0 (0.0)		685 (30.2)	589 (24.1)	
Green	1,029 (21.7)	1,029 (44.4)	0 (0.0)		582 (25.7)	433 (17.7)	
ABCDE (204)	4,295 (85.4)	1,929 (86.6)	2,071 (87.4)	0.4	2,139 (85.9)	2,124 (85.1)	0.4
SAMPLE (145)	1,015 (19.9)	456 (20.2)	520 (21.7)	0.2	368 (14.4)	647 (25.9)	< 0.001
Mode of transport (718)				< 0.001			< 0.001
Ground Ambulance	4,078 (90.3)	1,602 (84.3)	2,330 (98.0)		1,878 (86.9)	2,178 (93.7)	
HEMS	20 (0.4)	0 (0.0)	10 (0.4)		17 (0.8)	3 (0.1)	
Patient transport	69 (1.5)	61 (3.2)	7 (0.3)		12 (0.6)	57 (2.5)	
Police vehicle	35 (0.8)	19 (1.0)	0 (0.0)		29 (1.3)	4 (0.2)	
Other	315 (7.0)	219 (11.5)	31 (1.3)		224 (10.4)	82 (3.5)	
Decision on destination (109)				< 0.001			< 0.001
Remain at the scene	706 (13.8)	429 (18.9)	41 (1.7)		456 (17.8)	236 (9.4)	
Primary care/other care center	134 (2.6)	98 (4.3)	14 (0.6)		72 (2.8)	62 (2.5)	
Emergency department	3,832 (74.8)	1,642 (72.4)	2,032 (84.4)		1,927 (75.2)	1,875 (74.4)	
Fast-track, Hip fracture	334 (6.5)	29 (1.3)	303 (12.6)		22 (0.9)	312 (12.4)	
Other hospital/department facility	120 (2.3)	70 (3.1)	18 (0.7)		84 (3.3)	35 (1.4)	

^*1*^n (%) ^*2*^Pearson’s Chi-squared test; Fisher’s exact test ^*3*^Pearson’s Chi-squared test ^4^Missing data

Spinal motion restriction was the most common intervention at scene (6.6%), and the most common form of medical treatment was pain treatment with opiates (23.8%). Spinal motion restriction was also more associated with younger age (10.3% vs. 2.9%, p < 0.001) and more common among patients assessed with high acuity (10.4% vs. 2.7%, p < 0.001) (Table 5).

**Table 5:** EMS interventions in relation to assessed severity and age

	N = 5,235^*1*^	Low acuity, N = 2,315^*1*^	High acuity, N = 2,428^*1*^	p-value^*2*^	≤ 63 yrs, N = 2,613^*1*^	> 63 yrs, N = 2,567^*1*^	p-value^*2*^
Injury restriction, stabilisation							
Spinal motion restriction (106)	339 (6.6)	62 (2.7)	251 (10.4)	< 0.001	265 (10.3)	72 (2.9)	< 0.001
Fracture stabilisation/reposition (113)	256 (5.0)	82 (3.6)	164 (6.8)	< 0.001	139 (5.4)	117 (4.6)	0.2
Bleeding control, advanced interventions (94)							
	9 (0.2)	2 (0.1)	4 (0.2)	0.7	7 (0.3)	2 (0.1)	0.2
Pressure dressing	218 (4.2)	107 (4.7)	84 (3.5)	0.036	120 (4.7)	94 (3.7)	0.093
Pelvic stabilisation	16 (0.3)	0 (0.0)	11 (0.5)	0.001	12 (0.5)	4 (0.2)	0.049
Cardiopulmonary resucitation	27 (0.5)	0 (0.0)	0 (0.0)		15 (0.6)	11 (0.4)	0.5
Needle Thoracostomy	6 (0.1)	0 (0.0)	3 (0.1)	0.3	5 (0.2)	1 (0.0)	0.2
Cricothyrotomy	0 (0.0)	0 (0.0)	0 (0.0)		0 (0.0)	0 (0.0)	
Medical treatment yes (80)	2,187 (42.4)	691 (30.3)	1,376 (56.9)	< 0.001	1,033 (40.0)	1,143 (45.2)	< 0.001
Opioids	1,229 (23.8)	340 (14.9)	859 (35.5)	< 0.001	539 (20.9)	688 (27.2)	< 0.001
Ketamine	305 (5.9)	35 (1.5)	258 (10.7)	< 0.001	115 (4.5)	189 (7.5)	< 0.001
Acetaminophen	567 (11.0)	252 (11.0)	296 (12.2)	0.2	246 (9.5)	319 (12.6)	< 0.001
Sedatives	419 (8.1)	66 (2.9)	338 (14.0)	< 0.001	182 (7.1)	236 (9.3)	0.003
NSAID	12 (0.2)	6 (0.3)	2 (0.1)	0.2	10 (0.4)	2 (0.1)	0.023
Tranexamic acid	15 (0.3)	1 (0.0)	10 (0.4)	0.009	10 (0.4)	4 (0.2)	0.12
Oxygen	395 (7.7)	37 (1.6)	338 (14.0)	< 0.001	112 (4.3)	282 (11.2)	< 0.001
Infusion	480 (9.3)	66 (2.9)	397 (16.4)	< 0.001	122 (4.7)	355 (14.0)	< 0.001
Other drugs	795 (15.4)	267 (11.7)	459 (19.0)	< 0.001	439 (17.0)	350 (13.8)	0.002

^*1*^n (%) ^*2*^Pearson’s Chi-squared test; Fisher’s exact test ^3^Missing data

### Patient outcomes and treatment

The most common hospital treatment was fracture treatment1,220 (27.8%). followed by wound care 891 (20.3%3). Nearly half of the patients were X-rayed at the hospital (48.5%). Of those who were transported to the hospital, less than half (43.9%) were admitted to a hospital ward, and 2.6% were treated in an intensive care unit. The mortality rates after 2, 7 and 30 days were 1.1%, 1.8% and 3.4%, respectively. The mortality rate was significantly higher in the patient group > 63 years of age (Table 6).

**Table 6:** Patient outcome and treatment in relation to EMS assessed severity and age

	N = 5,235^*1*^	Low acuity, N = 2,315^*1*^	High acuity, N = 2,428^*1*^	p-value^*2*^	≤ 63 yrs, N = 2,613^*1*^	> 63 yrs, N = 2,567^*1*^	p-value^*2*^
Interventions/examinations (844)^3^							
Fracture treatment	1,220 (27.8)	397 (21.4)	782 (33.7)	< 0.001	416 (19.9)	802 (34.9)	< 0.001
Joint dislocation treatment	222 (5.1)	58 (3.1)	159 (6.9)	< 0.001	111 (5.3)	111 (4.8)	0.5
Wound care	891 (20.3)	371 (20.0)	475 (20.5)	0.7	442 (21.2)	449 (19.6)	0.2
Wound suturing	673 (15.3)	290 (15.6)	348 (15.0)	0.6	318 (15.2)	355 (15.5)	0.8
Antibiotics	314 (7.2)	85 (4.6)	210 (9.1)	< 0.001	119 (5.7)	195 (8.5)	< 0.001
CT scan	1,760 (40.1)	595 (32.1)	1,110 (47.9)	< 0.001	786 (37.6)	972 (42.3)	0.002
X-ray	2,097 (47.8)	788 (42.5)	1,266 (54.6)	< 0.001	747 (35.8)	1,348 (58.7)	< 0.001
Other interventions/care	1,712 (39.0)	679 (36.6)	940 (40.5)	0.009	841 (40.3)	867 (37.8)	0.088
Transfusion dependent	208 (4.7)	24 (1.3)	175 (7.5)	< 0.001	27 (1.3)	181 (7.9)	< 0.001
Operation fracture	813 (18.5)	173 (9.3)	618 (26.6)	< 0.001	243 (11.6)	569 (24.8)	< 0.001
Organ dysfunction OR	56 (1.3)	6 (0.3)	39 (1.7)	< 0.001	29 (1.4)	27 (1.2)	0.5
Other complication OR	185 (4.2)	36 (1.9)	131 (5.6)	< 0.001	89 (4.3)	96 (4.2)	0.9
Admission to hospital (880)	1,910 (43.9)	518 (28.2)	1,324 (57.4)	< 0.001	568 (27.6)	1,339 (58.5)	< 0.001
ICU care (680)	117 (2.6)	14 (0.7)	88 (3.8)	< 0.001	64 (2.9)	53 (2.3)	0.2
Days of admission (1,044)	1.0 (0.0, 5.0)	0.0 (0.0, 1.0)	1.0 (0.0, 7.0)	< 0.001	0.0 (0.0, 1.0)	2.0 (0.0, 8.0)	< 0.001
Secondary transport to other hospital (890)	294 (6.8)	82 (4.5)	198 (8.6)	< 0.001	104 (5.1)	189 (8.3)	< 0.001
Discharged to (1,220)				< 0.001			< 0.001
Residential home	3,346 (83.3)	1,444 (89.2)	1,787 (79.4)		1,751 (95.1)	1,592 (73.4)	
Nursing home	469 (11.7)	129 (8.0)	334 (14.8)		10 (0.5)	459 (21.2)	
Other hospital/rehab clinic	129 (3.2)	36 (2.2)	86 (3.8)		67 (3.6)	61 (2.8)	
Deceased in hospital	71 (1.8)	10 (0.6)	44 (2.0)		13 (0.7)	58 (2.7)	
All-cause mortality (143)							
2 day mortality	58 (1.1)	5 (0.2)	14 (0.6)	0.051	26 (1.0)	32 (1.3)	0.4
7 day mortality	94 (1.8)	12 (0.5)	40 (1.7)	< 0.001	28 (1.1)	66 (2.6)	< 0.001
30 day mortality	173 (3.4)	33 (1.5)	97 (4.1)	< 0.001	29 (1.1)	144 (5.6)	< 0.001

^*1*^n (%); Median (IQR) ^*2*^Pearson’s Chi-squared test; Wilcoxon rank sum test ^3^Missing data

## Discussion

This study showed that the median age of injured patients treated by EMS was 63 years, and the largest age group was between 18 and 64 years. Women were most common in the oldest age groups. This agrees well with data from a previous Norwegian study that included patients who were subject to trauma activation in hospitals [4].

In this study, injuries that occurred in residential areas accounted for 55% of injuries and traffic accidents were surprisingly few, and considering the potential energy that could lead to severe injury among injuries sustained by traffic, the percentage of high acuity in this group was relatively low compared with injuries not related to traffic. However, in Sweden, large resources have been invested in reducing the number of people injured in traffic. The Swedish Transport Administration leads the work towards better traffic safety. This work includes a zero vision, where the goal is that no one should die or be seriously injured in traffic [16]. Among other things, the strategies have included improved education, application and structural improvements, such as the installation of medians barriers, roundabouts, speed humps and pedestrians’ islands. Despite all these measures, the number of seriously injured and killed people in traffic has not decreased in the last 10 years [17]. Compared to the cost of reducing traffic accidents, fall prevention is relatively cheap. According to a report from the Swedish National Board of Health and Welfare [18], fall prevention is very cost-effective. Despite this, limited work has been done on fall prevention in Swedish municipalities. Research has shown that multifaceted programmes in fall prevention can have a positive effect in reducing the number of falls in the residential environment [19]. Fall-prevention programmes can include interventions such as home risk assessments and behavioural strategies, including fall-prevention education, exercise programmes and medication reviews.

In the present study, wounds, hematomas and fractures were the most common injuries, this is well in line with previously reported data from Swedish hospitals [5]. The older patients had more deviating vital parameters with lower oxygen saturation and higher blood pressure and serum glucose levels. It could certainly be due to changes in the vital parameters even before the accident but could also be a sign that older patients suffer worse from injuries compared to younger patients.

The most common prehospital intervention was medical treatment against pain. A large proportion (74.9%) of the patients in the study had pain of varying degrees, but only 40.7% received any form of medical pain treatment. This might be an area of improvement. The most common treatment was the use of opioids, acetaminophen and ketamine. Both opioids and ketamine have been documented to have a positive effect on trauma pain in the prehospital setting, but ketamine is likely better than opiates, with better reduction of pain and fewer unwanted effects such as nausea, hypotension and impaired respiration [20, 21].

Despite the fact that nearly one out of five in this study population had some sort of fracture, only one out of twenty received fracture stabilisation and/or reposition in the prehospital setting. The reason may be that many patients are older. There are challenges in the prehospital assessment of elderly trauma patients. Among other things, it is more difficult to assess the mechanism of injury and its impact on the patient, as many have been exposed to low-energy trauma [22]. This can lead to injuries such as fractures being missed in the prehospital setting and thus not receiving treatment. The assessment of older patients can be affected by co-morbidities, such as delirium or dementia. Previous studies have shown that older patients are more likely to be left at home after injuries than younger patients [22]. In this study, half as many patients in the older group were left at home compared to younger patients. This can be considered a positive result of the study.

Patients over the age of 63 had a significantly increased in-hospital mortality after the injury. Of course, this may have natural causes and this study cannot determine if the cause is related to the injury or if there are other causes. However, trauma systems in Sweden and around the world mostly focus on high-energy trauma. The most common trauma activation criteria in Swedish hospitals [23] are based on recommendations from the American College of Surgeons [24] and the Centers for Disease Control and Prevention [25]. The guidelines are focused on high-energy trauma. The American guidelines include elderly patients and state that other limits for vital parameters apply to this patient group and that even low-energy trauma can include serious injuries that require transportation to a trauma hospital. The Swedish guidelines only state that physicians at the receiving hospital should be contacted by the EMS in the case of low-energy trauma in elderly patients. This fact probably has the consequence that trauma activation is rarely activated in this patient group. Under-triage of older trauma patients is an international problem. A British study [26] found that older trauma patients were less likely than younger trauma patients to receive trauma team activation. The study also found an under-triage rate of 65.2% among older trauma patients. In an American study [27], the research group searched for predictors for under-triage among trauma patients over 70 years. They found that age over 80 was a predictor for under-triage, whereas the type of injury did not affect the triage level. Specific prehospital triage systems for older trauma patients have increased the sensitivity but unfortunately have not shown any major difference in mortality [27]. These results indicate that under-triage among older trauma patients remains even after the introduction of specific triage systems for elderly trauma patients. Furthermore, in this study, there were signs of under-triage among patients over 63 years of age. Although this patient group has significantly higher hospital admission and mortality rates, fewer were given the highest priority by the dispatch centre, fewer were triaged as red by the EMS and fewer were transported by HEMS.

### Strengths and limitations

One of the study’s strengths is that data were collected from all EMS and hospital organisations in a large region in Sweden. A relatively large number of EMS records were randomly selected, manually reviewed and matched against hospital records by experienced ambulance nurses. The study cohort is well-defined and has not previously been described in these terms.

Missing data due to the design of the study is a limitation. One reason might depend on the dual documentation with both paper and electronic documentation (i.e., data such as vital signs might have been measured and documented on paper but not electronically registered). A major limitation is the limited information on the patient’s pain intensity before and after pain management, according to NRS. It is reasonable to assume that the result depends on several factors, such as many patients being acutely cognitively impaired or suffering from dementia. Behavioural pain assessment tools are feasible alternatives to numerical scales to improve the assessment of patients’ pain. However, information about medical treatment was, to a great extent, electronically registered. Follow-up by means of electronic records is an area of improvement to increase knowledge of the quality of care in the EMS. Another weakness is the lack of information on the prehospital death rate.

## Conclusion

Among primary EMS assignments in southwestern Sweden, 17% were caused by injury equally distributed between women and men. More than half of these cases were caused by low-energy falls, and the most common trauma location was a residential area. The majority of the victims had pain upon arrival of the EMS, and a large proportion appeared to have severe pain.

## Data Availability

The datasets generated and analysed during the current study are not publicly available due to participant anonymity issues but are available from the corresponding author on reasonable request.

## List of Abbreviations

EMDC: Emergency Medical Dispatch Centre
EMS: Emergency Medical Service
ESS: Emergency Signs and Symptoms
HEMS: Helicopter Emergency Medical Services
NRS: Numeric Rating Scale
RETTS: Rapid Emergency Triage and Treatment System
